# Characterization of a Novel Mycovirus from the Phytopathogenic Fungus *Botryosphaeria dothidea*

**DOI:** 10.3390/v14020331

**Published:** 2022-02-06

**Authors:** Yanfen Wang, Hang Zhao, Jiayuan Cao, Xinming Yin, Yashuang Guo, Lihua Guo, Haiyan Wu, Meng Zhang

**Affiliations:** 1 School of Plant Protection, Henan Agricultural University, Zhengzhou 450002, China; wyfhist@163.com (Y.W.); zhaohang96@foxmail.com (H.Z.); jiayuan_Cao@126.com (J.C.); xmyin@henau.edu.cn (X.Y.); guoyashuang@henau.edu.cn (Y.G.); 2 State Key Laboratory for Biology of Plant Diseases and Insect Pests, Institute of Plant Protection, Chinese Academy of Agricultural Sciences, Beijing 100193, China; guolihua72@yahoo.com; 3 Analytical Instrument Center, Henan Agricultural University, Zhengzhou 450002, China

**Keywords:** *Botryosphaeria dothidea*, Botryosphaeria dothidea partitivirus 2 (BdPV2), *Gammapartitivirus*

## Abstract

*Botryosphaeria dothidea* is, globally, one of the most economically important phytopathogenic fungi worldwide, causing the canker and dieback of fruit trees. An increasing number of viruses infecting *B. dothidea* have lately been reported, several of which could confer hypovirulence. In this study, isolated from strain ZM170285-1 of *B. dothidea*, a novel double-stranded RNA (dsRNA) mycovirus, tentatively named Botryosphaeria dothidea partitivirus 2 (BdPV2), was identified well. The BdPV2 harbored three dsRNA segments (1–3) with lengths of 1751, 1568, and 1198 bp, which encoded an RNA-dependent RNA polymerase (RdRp), a capsid protein (CP), and a hypothetical protein of unknown function, respectively. BLASTp searches revealed that the predicted protein sequences of dsRNA1 and dsRNA2 had the highest identities (74.95% and 61.01%) with the corresponding dsRNAs of Penicillium stoloniferum virus S (PsV-S), whereas dsRNA3 shared the highest identity (32.95%) with the dsRNA3 of Aspergillus ochraceous virus 1 (AoV1). Phylogenetic analysis indicated that BdPV2 belonged to the *Gammapartitivirus* genus and *Partitiviridae* family. To our knowledge, this is the first report of a *Gammapartitivirus* in *B. dothidea*.

## 1. Introduction

Mycoviruses are widely distributed and reported in nearly all major groups of fungi [1,2,3]. Some mycoviruses can cause the hypovirulence of hosts and, therefore, be used as biocontrol agents for certain fungal diseases [4,5,6]. Although most mycoviruses had double-stranded RNA (dsRNA) or positive-sense single-stranded RNA (+ssRNA) genomes, several negative-sense single-stranded RNA (−ssRNA) or single-stranded DNA (ssDNA) were described [7,8,9,10]. Mycoviruses with dsRNA genomes were classified into seven families: *Chrysoviridae*, *Partitiviridae*, *Reoviridae*, *Megabirnaviridae*, *Totiviridae*, *Quadriviridae*, and *Endornaviridae* [11]. Members of the *Partitiviridae* family generally have two or three genome segments (1.4 to 2.4 kbp in length) encoding an RNA-dependent RNA polymerase (RdRp), a capsid protein (CP), and a hypothetical protein with unknown function, respectively [12]. Up to now, five genera in this family have been approved by the International Committee for the Taxonomy of Viruses (ICTV), namely, *Alphapartitivirus*, *Betapartitivirus*, *Cryspovirus*, *Deltapartitivirus*, and *Gammapartitivirus* [13]. Recently, however, two additional genera, Epsilsonpartitivirus and Zetapartitivirus, were proposed [14]. Even if most partitiviruses exhibited no obvious effects on their host fungi [12], a few exceptions could interfere with the natural physiology, including morphology, toxin production, and hypovirulence [15,16,17].

*Botryosphaeria dothidea* (Moug.: Fr.) Ces. and De Not. is a ubiquitous phytopathogen with a broad host range, including pear, poplar, apple, walnut, and jujube trees [18,19,20], mainly causing fruit rot, branch dieback, and stem warts [21]. Several mycoviruses are present in *B. dothidea* isolates infecting pears and apples but not found in *B. dothidea* isolates infecting walnuts. To date, 11 mycoviruses from *B. dothidea* have been identified belonging to the families *Partitiviridae*, *Totiviridae*, *Chrysoviridae*, *Narnaviridae*, *Botourmiaviridae*, and *Mitoviridae* [6,22,23,24,25,26,27,28,29], the newly proposed family Fusariviridae [25], and an unassigned group [29]. Of these mycoviruses, Botryosphaeria dothidea chrysovirus 1 (BdCV1), Botryosphaeria dothidea RNA virus 1 (BdRV1), Bipolaris maydis botybirnavirus 1 strain BdEW220 (BmRV1-BdEW220) could attenuate the virulence of *B. dothidea* [6,28,29], while the others did not.

In this study, a novel dsRNA virus from strain ZM170285-1 of *B. dothidea* was identified and characterized. This is the second partitivirus discovered in *B. dothidea*, tentatively named Botryosphaeria dothidea partitivirus 2 (BdPV2). BdPV2 could be transmitted via asexual sporulation at 100%.

## 2. Materials and Methods

### 2.1. Fungal Strains and Culture Conditions

Strains ZM170285-1 and ZM180088 of *B. dothidea* were isolated from walnut trunks collected in Sanmenxia, Henan province, China. Virus-infected strain ZM170285-1 had an abnormal colony morphology. Protoplast regeneration, hyphal tipping, and ribavirin treatment were used to eliminate the mycoviruses in strain ZM170285-1 [30]. Virus-free strain ZM180088 was used as a control for evaluating biological characteristics. Both strains were cultured on potato dextrose agar (PDA) plates at 25 °C and stored in sterile 30% glycerol solution at 4 °C.

### 2.2. Extraction and Purification of dsRNA

Mycelia of the *B. dothidea* strains were cultured on cellophane membranes laid on PDA plates (9 cm in diameter) for 5 days at 25 °C. Fresh mycelia were harvested and ground into a fine powder in liquid nitrogen. The extraction and purification of dsRNA were performed as previously described [6]. dsRNA samples were treated with both DNase I and S1 nuclease (TaKaRa, Dalian, China). Purified dsRNA elements were separated in 1.2% agarose gel at 120 V and visualized in a UV transilluminator after staining with ethidium bromide (EB).

### 2.3. cDNA Synthesis and Cloning

cDNA synthesis and molecular cloning were performed using random primers (5′-CGATCGATCATGATGCAATGCNNNNNN-3′) as previously described [28]. PCR amplicons were ligated into the pMD18-T vector (TaKaRa, Dalian, China) and then transformed into competent *Escherichia coli* DH5α cells. Sequences gaps between clones were determined by reverse transcription-PCR (RT-PCR) using specific primers designed on the basis of obtained cDNA sequence. The terminal sequences of each dsRNA were determined [6].

### 2.4. Sequence Analysis

Sequence searches were conducted using the BLASTX or BLASTP program in the NCBI databases (URL). Multiple sequence alignments were performed on the basis of conserved amino acid sequences using MAFFT software [31]. The aligned sequences were shaded using the BoxShade website (https://embnet.vital-it.ch/software/BOX_form.html, accessed on 6 January 2020). ORFs were predicted using ORFfinder (https://www.ncbi.nlm.nih.gov/orffinder/, accessed on 6 January 2020). Phylogenetic trees were constructed using maximum likelihood with a Jones–Taylor–Thornton (JTT) model using MEGA7 software version 7.0 [32]. Bootstrap values were evaluated with 1000 replicates.

### 2.5. Transmission Assay

Conidia production of the strain ZM170285-1 was induced on PDA plates under alternating light/dark (16 h:8 h) cycles [18]. We individually selected 66 single-conidia isolates to be used for assessing the presence of dsRNAs. Hyphal anastomosis was used to investigate the horizontal transmission of dsRNAs between strains ZM170285-1 and ZM180088. Virus-free (BdPV2^−^) strain ZM180088 (as the receptor) and virus-infected (BdPV2^+^) strain ZM170285-1 (as the donor) were dual cultured on PDA plates in the dark for 10 days at 25 °C. Ten mycelia agar plugs were obtained from the colony margin of recipients (the farthest site from strain ZM180088) and transferred to a fresh PDA plate for purification with single hyphal tips. The viral transmission was assessed on the basis of RT-PCR amplification and dsRNA extraction.

### 2.6. Biological Testing

Mycelia agar plugs from the colony margin of 3-day-old cultures were inoculated onto fresh PDA medium for subculture. The colony diameters of each strain were measured daily for 3 days, and their mycelia growth rates were calculated. The virulence of the fungal strain was determined according to a previously described method [21]. Briefly, freshly excised mycelia agar plugs of each isolate were inoculated onto four detached 1-year-old stems of walnut trees. Vaccination sites were covered with sterilized moist cotton balls to maintain 90% relative humidity for 2 days at 30 °C. Lesions induced by the fungus were measured and photographed 10 days post inoculation (dpi). Each treatment described above was performed more than three times.

## 3. Results

### 3.1. Genetic Analysis of BdPV2

**Figure 1:**
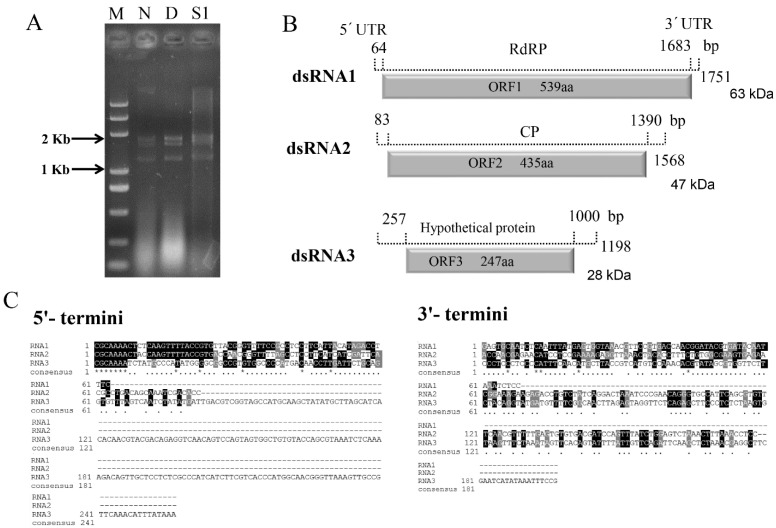
Genomic organization of BdPV2. (**A**) Lane M, DL5000 DNA marker; Lane N, dsRNA extracted from strain ZM170285-1; Lanes D and S1, dsRNA was treated with DNase I and S1 nuclease. (**B**) Schematic representation of genomic organization of BdPV2. (**C**) Multiple sequence alignment of 5′- and 3′-UTRs of BdPV2. *, conserved amino acid residues.

Complete dsRNA sequences were obtained by using RT-PCR amplification combined with the RLM-RACE procedure. The full lengths of cDNAs of dsRNAs 1 to 3 are 1751, 1568, and 1198 bp, respectively (Figure 1A,B), which were submitted to GenBank under accession numbers MZ044010 to MZ044012. ORFfinder prediction revealed that each of the 3 dsRNAs contained one ORF.

The largest dsRNA1 (49.8% G+C content) contained one large ORF from nucleotide (nt) 64 to 1683, encoding a protein (P1) of 539 amino acids with a predicted molecular mass (Mr) of 63 kDa (Figure 1B). BLASTp searched of P1 revealed that it shares the highest identity (74.95%, *E*-value 0, coverage 100%) with the RNA-dependent RNA polymerase (RdRp) of Penicillium stoloniferum virus S (PsV-S) and Penicillium brevicompactum partitivirus 1 (PbPV1). P1 also shared similar identities (67.53–74.21%) to the RdRps of Penicillium digitatum partitivirus 1 (PdPV1), Aspergillus ochraceous virus 1 (AoV1), Verticillium dahliae partitivirus 1 (VdPV1), Aspergillus fumigatus partitivirus 1 (AfuPV-1), and Gremmeniella abietina RNA virus MS1 (GaRV-MS1) (Table 1). Similarly, dsRNA2 (56.57% G+C content) contained a single ORF from nt 83 to 1390 encoding a 435 aa protein (P2) with a predicted Mr of 47 kDa (Figure 1B). BLASTp analysis revealed that P2 shared the highest identity (61.01%, *E*-value 0, coverage 100%) with the capsid protein (CP) of PsV-S and high identities (52.41–60.00%) with the CPs of the other members of the *Partitiviridae* family (Table 1). The smallest dsRNA3 (48.1% G+C content) contained a single ORF at nt 257–1000 encoding a 247–aa protein (P3) with a predicted Mr of 28 kDa (Figure 1B). P3 shared the highest identity (32.95%, *E*-value 1 × 10^−28^, coverage 99%) with a protein of unknown function encoded by dsRNA3 of AoV1. Additionally, P3 shared identities with the putative proteins encoded by dsRNA3 of AfuPV-1 A (37.07%, *E*-value 5 × 10^−26^, coverage 79%) and GaRV-MS1 (31.58%, *E*-value 1 × 10^−20^, coverage 79%) (Table 1).

The 5′-untranslated regions (UTRs) of the plus strands of segments dsRNAs 1 to 3 are, respectively, 63, 82, and 256 bp long and share conserved nucleotides (CGCAAA) (Figure 1C). The corresponding 3′-UTRs are 68, 178, and 198 bp long, respectively, and share little sequence conservation (Figure 1C). According to the ICTV, the sequence identity of CP ≤ 80% and RdRp ≤ 90% are required to define a new species in the *Gammapartitivirus* genus [11]. Thus, results indicated that dsRNAs 1 to 3 represent the genomic components of a novel virus tentatively named Botryosphaeria dothidea partitivirus 2 (BdPV2), belonging to *Gammapartitivirus* within the *Partitiviridae* family.

### 3.2. Phylogenetic Analysis of BdPV2

**Figure 2:**
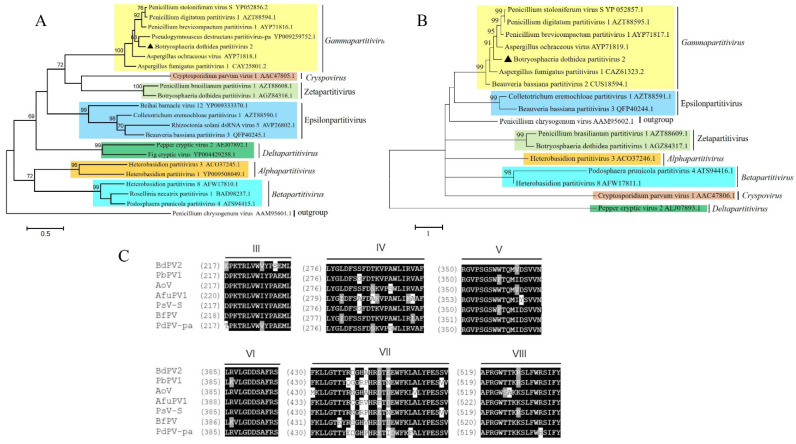
Phylogenetic analysis of BdPV2. Maximum-likelihood (ML) phylogenetic analysis based on amino acid sequences of (**A**) RdRp and (**B**) CP of BdPV2 and other selected members of seven genera of the *Partitiviridae* family. Phylogenetic tree was generated using MEGA 7 with 1000 bootstrap replicates. Triangle indicates position of BdPV2. (**C**) Multiple alignments of amino acid sequences of RdRp motifs of BdPV2 and other selected mycoviruses in *Partitiviridae* family. Six motifs (III–VIII) detected in conserved RdRp sequence. Similar amino acid residues are shaded in black.

Phylogenetic trees were established on the basis of amino acid sequences of RdRp and CP from BdPV2 and those of partitiviruses selected from all genera (including newly proposed ones) of the *Partitiviridae* family, respectively. Results showed that BdPV2 and related partitiviruses (PsV-S, PdPV1, PbPV1, AoV1, and AfuPV-1) were clustered into the *Gammapartitivirus* cluster (Figure 2A,B). Multiple sequence alignment based on the amino acid sequence of the encoded RdRp revealed six conserved motifs III–VIII in BdPV2 within members of the *Partitiviridae* family (Figure 2C).

### 3.3. Transmission Assay of BdPV2

In total, 66 single-conidia derived from *B. dothidea* strain ZM170285-1 were all infected with BdPV2. To evaluate whether BdPV2 can be horizontally transmitted between *B. dothidea* strains, strain ZM170285-1 (BdPV2-infected, BdPV2^+^) was dual cultured with strain ZM180088 (BdPV2-free, BdPV2^−^) on PDA plates. The infection status of receptor strain ZM180088 was assessed by RT-PCR amplification 10 days after dual culture using RNA isolated from mycelia disks excised from the colony margins of the receptor strain (the farthest site from strain ZM180088). None of the 10 tested derivative sub-strains was infected with BdPV2, indicating no viral transfer.

### 3.4. Effects of BdPV2 on B. dothidea

**Figure 3:**
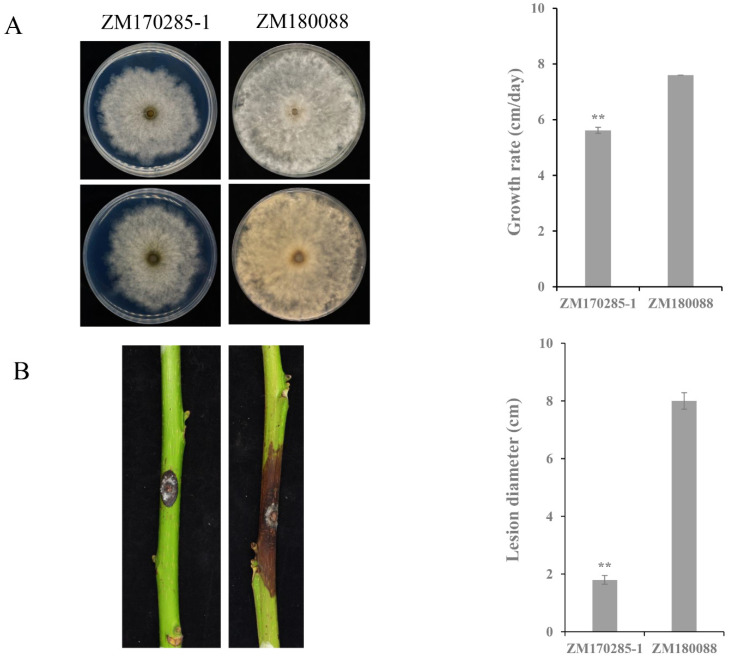
Biological characteristic comparison between strains ZM170285-1 and ZM180088. (**A**) Colony morphology and growth rates of strains ZM170285-1 and ZM180088 grown at 25 °C for 3 days on PDA medium. (**B**) Pathogenicity assay and lesion diameters of two strains grown on detached walnut stems at 10 days post inoculation at 30 °C, and approximately 90% relative humidity. **, indicates a significant difference (*p* < 0.01) between strains ZM170285-1 and ZM180088.

To obtain the virus-free BdPV2 isogenic lineage, a variety of methods, including hyphal tip culture, protoplast regeneration, and ribavirin treatment, have been tried, but all failed. Meanwhile, to check the effects of BdPV2 on the biological characteristics of *B. dothidea*, the colony morphology and growth rate of strains ZM170285-1 (BdPV2^+^) and ZM180088 (BdPV2^−^) were compared. Strain ZM180088 grew rapidly with a growth rate of 3.0 cm/d and developed dense mycelia, while strain ZM170285-1 grew slowly with a growth rate of 1.8 cm/d and exhibited abnormal morphologies with irregular colony margins (Figure 3A). As inoculated on branches, strain ZM170285-1 induced small necrotic lesions (approximately 1.8 cm) around the inoculation point of the walnuts, while strain ZM180088 induced obviously long (approximately 8 cm) and typical brown necrosis lesions (Figure 3B). These results indicate that strain ZM170285-1 is weakly virulent.

## 4. Discussion

To date, only a few *B. dothidea* isolates from pear or apple have been reported to possess several mycoviruses [27]. However, there is no research on mycoviruses of *B. dothidea* strains that infected any other fruit trees. In this study, from *B. dothidea* strain ZM170285-1 (causing walnut dieback), we isolated and characterized novel mycovirus BdPV2 with three dsRNA segments that encodes an RdRp, a CP, and a hypothetical protein of unknown function. This genomic organization of BdPV2 is in full accordance with mycoviruses AoV1, AfuPV-1 A, and GaRV-MS1 [37,39,40], but quite different from PsV-S, PbPV1, PdPV1, and VdPV1 [33,34,35,36], which all have just two dsRNA segments encoding an RdRp and a CP. The 5′-UTRs of dsRNAs 1 to 3 of BdPV2 showed a conserved nucleotide motif (CGCAAA), which is a feature of multi-component RNA viruses [12].

On the basis of phylogenetic analysis, known viruses of the *Gammapartitivirus* genus were separated into several different clades (Figure 3A,B). BdPV2 was more closely related to AoV1 that could infect the plant pathogenic fungus *Aspergillus ochraceus* [37]. The amino acid sequences encoded by dsRNAs 1 to 3 of BdPV2 shared 72.9%, 57.9%, and 32.9% identities, respectively, with the corresponding dsRNAs of AoV1. The 5′-terminal sequences of dsRNAs 1 to 3 shared 59.7%, 51.1%, and 42.6% identities, respectively, with the corresponding sequences of AoV1 [37]. The conserved regions near the 5′ termini of viral genomic RNA may be involved in viral packaging and/or replication [41].

Most mycoviruses can be transmitted vertically via fungal spores or horizontally via hyphal anastomosis [10]. In nature, vegetative incompatibility reduces or abolishes mycoviral transmission between fungal strains, whereas vegetatively compatible fungi can easily transmit and exchange mycoviruses [42]. Both SsHADV-1 and SsPV1 were reported to be able to transmit between different vegetatively compatible individuals [5,43]. However, Wu et al. (2017) discovered that SsMYRV4 could not only be transmitted among vegetatively incompatible groups but also promote the transmission of other viruses [44]. In this study, BdPV2 could not be transmitted to the *B. dothidea* strain ZM180088 after dual culture, which was similar to BdPV1 [6]. Another report found that BmBRV1-BdEW220 could be transmitted among different strains derived from a single conidium but not transferred to strains of different origins [28]. Therefore, further studies are required to assess if BdPV2 can be transferred to strains from similar origins. As anticipated for partitiviruses [45], BdPV2 was easily transmissible via asexual conidia. To obtain the virus-free ZM170285-1 isogenic lineage, we attempted to eliminate BdPV2 dsRNA from infected cultures failed, including hyphal tip culture, protoplast regeneration, and ribavirin treatment, suggesting that this virus may have evolved effective strategies in *B. dothidea*.

Partitiviruses generally cause latent infections and seldom alter the biological traits of their fungal hosts [16,17]. Nevertheless, some partitiviruses could cause abnormal phenotypes or reduced virulence of their hosts. For instance, SsPV1 not only caused the slow growth and abnormal colony morphology of *S. sclerotiorum* but also reduced its virulence and sclerotium production [43]. AfuPV1 infection elicited the abnormal colony phenotype, slow growth, and lighter pigmentation [38]. In the current study, there were significant differences between the growth rate and virulence of the BdPV2-infected strain ZM170258-1 and the BdPV2-free strain ZM180088. However, due to the absence of virus-free isogenic lineage, it is difficult to obtain details of the interaction between BdPV2 and *B. dothidea*. Therefore, it is not clear whether the morphological and weak pathogenicity of *B. dothidea* strain ZM170285-1 is associated with BdPV2 infection, and further investigation is needed to explore its potential for use in the biological control of diseases caused by *B. dothidea*.

## Figures and Tables

**Table 1:** Information of BLASTp search results of Botryosphaeria dothidea partitivirus 2 (BdPV2).

Virus Name	Segment Number	ORF (aa)	Accession Number	Query Cover (%)	Identity (%)	*E*-Values	Reference
Penicillium stoloniferum virus S (PsV-S)	1	539	YP_052856	100	74.95	0	[33]
	2	434	YP_052857	100	61.01	0	
Penicillium brevicompactum partitivirus 1 (PbPV1)	1	539	AYP71816	100	74.95	0	[34]
	2	433	AYP71817	100	60.00	0	
Penicillium digitatum partitivirus 1 (PdPV1)	1	539	AZT88594	100	74.21	0	[35]
	2	434	AZT88595	100	59.86	0	
Verticillium dahliae partitivirus 1 (VdPV1)	1	539	YP_009164038	100	67.53	0	[36]
	2	436	YP_009164039	99	56.68	6 × 10^−168^	
Aspergillus ochraceous virus 1 (AoV1)	1	539	YP_009665972	100	72.91	0	[37]
	2	433	AYP71819	100	57.93	2 × 10^−160^	
	3	293	AYP71820	99	32.95	1 × 10^−28^	
Aspergillus fumigatus partitivirus 1 (AfuPV-1) AfuPV-1 A dsRNA3	1	547	CAY25801	100	67.53	0	[38]
	2	368	CAZ61323	100	55.63	5 × 10^−170^	
	3	258	CAA7351346	79	37.07	5 × 10^−26^	[39]
Gremmeniella abietina RNA virus MS1 (GaRV-MS1)	1	539	AII16004	99	70.87	0	[40]
	2	443	AII16002	99	52.41	3 × 10^−155^	
	3	237	NP_659029	79	31.58	1 × 10^−20^	

## Data Availability

Not applicable.
